# Ecto-5′-Nucleotidase: A Candidate Virulence Factor in *Streptococcus sanguinis* Experimental Endocarditis

**DOI:** 10.1371/journal.pone.0038059

**Published:** 2012-06-07

**Authors:** Jingyuan Fan, Yongshu Zhang, Olivia N. Chuang-Smith, Kristi L. Frank, Brian D. Guenther, Marissa Kern, Patrick M. Schlievert, Mark C. Herzberg

**Affiliations:** 1 Department of Diagnostic and Biological Sciences, School of Dentistry, University of Minnesota, Minneapolis, Minnesota, United States of America; 2 Department of Microbiology, University of Minnesota Medical School, Minneapolis, Minnesota, United States of America; 3 Mucosal and Vaccine Research Center, Minneapolis Veterans Affairs Medical Center, Minneapolis, Minnesota, United States of America; Centers for Disease Control & Prevention, United States of America

## Abstract

*Streptococcus sanguinis* is the most common cause of infective endocarditis (IE). Since the molecular basis of virulence of this oral commensal bacterium remains unclear, we searched the genome of *S. sanguinis* for previously unidentified virulence factors. We identified a cell surface ecto-5′-nucleotidase (Nt5e), as a candidate virulence factor. By colorimetric phosphate assay, we showed that *S. sanguinis* Nt5e can hydrolyze extracellular adenosine triphosphate to generate adenosine. Moreover, a *nt5e* deletion mutant showed significantly shorter lag time (P<0.05) to onset of platelet aggregation than the wild-type strain, without affecting platelet-bacterial adhesion in vitro (P = 0.98). In the absence of *nt5e*, *S. sanguinis* caused IE (4 d) in a rabbit model with significantly decreased mass of vegetations (P<0.01) and recovered bacterial loads (log_10_CFU, P = 0.01), suggesting that Nt5e contributes to the virulence of *S. sanguinis* in vivo. As a virulence factor, Nt5e may function by (i) hydrolyzing ATP, a pro-inflammatory molecule, and generating adenosine, an immunosuppressive molecule to inhibit phagocytic monocytes/macrophages associated with valvular vegetations. (ii) Nt5e-mediated inhibition of platelet aggregation could also delay presentation of platelet microbicidal proteins to infecting bacteria on heart valves. Both plausible Nt5e-dependent mechanisms would promote survival of infecting *S. sanguinis*. In conclusion, we now show for the first time that streptococcal Nt5e modulates *S. sanguinis*-induced platelet aggregation and may contribute to the virulence of streptococci in experimental IE.

## Introduction

*Streptococcus sanguinis*, an oral commensal bacterium, is the most common cause of infective endocarditis (IE) [1], [2]. IE is characterized by the formation of septic thrombi or vegetations on the heart valves. The development of IE involves bacterial adherence, persistence and growth. Adherence is mediated by bacterial surface molecules and host tissues [3]–[6]. During transient bacteremia, adherent *S. sanguinis* attract monocytes and induce them to produce tissue factor activity and cytokines, which activate the coagulation cascade. The valve infection by *S. sanguinis* can also be mediated by direct interaction with platelets in the vegetation [3], [4]. In an animal model, *S. sanguinis* adherence to platelets and induction of aggregation into an in vitro thrombus correlate with the increased severity of IE [4]. How *S. sanguinis* resists monocyte engulfment, platelet killing to eventually drive the formation of septic vegetations on heart valves remains unclear.

Among the agents known to modulate platelet aggregation are adenine nucleotides, which are released from platelets into the surrounding microenvironment upon stimulation with pro-thrombotic agents [7]. Indeed, we have previously shown that dense granules containing ATP and ADP are released from platelets in response to *S. sanguinis* [8]. These nucleotides can be hydrolyzed by *S. sanguinis* cell wall nucleotidase activity to modulate platelet aggregation [9], [10]. In an animal model of IE, sortase A-anchored cell surface proteins were suggested to contribute to bacterial virulence [11], [12]. To identify the candidate enzyme, we searched the genome of *S. sanguinis* SK36, and four genes containing LPxTG motifs were found that encode putative cell surface nucleotidases [13]. The four predicted nucleotidase products would be anchored to the cell wall by a sortase A-dependent mechanism [14]. One of them, ecto-5′-nucleotidase (Nt5e), is a putative surface-located enzyme that could hydrolyze extracellular ATP, ADP, AMP to adenosine [15]. Since ADP is an agonist [16] and adenosine is a potent platelet aggregation antagonist [17], 5′-nucleotidase activities on the surface of *S. sanguinis* may modulate *S. sanguinis*-induced platelet aggregation and the severity of IE.

During bacterial infection, ATP and adenosine are also important immune regulators. For example, extracellular ATP can trigger monocyte release of proinflammatory cytokines such as IL-1β and IL-18 [18]. In contrast, adenosine is a potent immunosuppressive molecule, which inhibits IL-12 production, and increases IL-10 in monocytes [19], [20]. Successful host defense balances pro- and anti-inflammatory mediators. Hence, the consumption of ATP as well as generation of adenosine by *S.* *sanguinis* Nt5e could influence the immune response during infection. We, therefore, hypothesize that Nt5e contributes to the virulence of *S. sanguinis* in IE.

To identify and characterize the enzymatic activities of Nt5e, we have generated a *nt5e* deletion mutant (Δ*nt5e*) by allelic exchange and also complemented this strain to obtain *nt5e*+. We showed *S.* *sanguinis* NT5E hydrolyzes extracellular ATP to generate adenosine. Using platelet aggregation and platelet-bacterial adhesion assays, we also showed that Nt5e delayed *S. sanguinis*-induced human platelet aggregation without affecting platelet-bacterial adhesion. Finally, Nt5e was determined to be a plausible virulence factor in a rabbit endocarditis model.

## Results

### Identification of the *S. sanguinis* Protein Possessing Cell Surface Nucleotidase Activity

**Figure 1:**
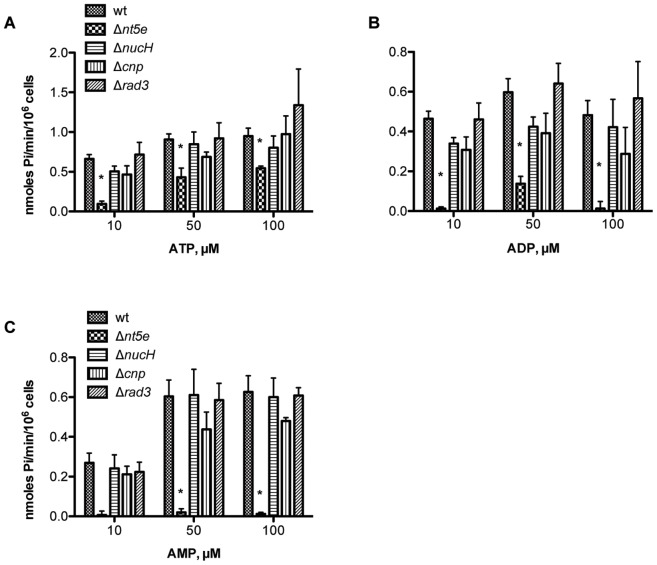
*nt5e* confers Nt5e activity on *S. sanguinis* SK36 whole cells. Nt5e activity was measured by the release of inorganic phosphate (Pi) from adenine nucleotides. (A), (B), and (C) were showed as enzyme velocity vs. concentration of ATP, ADP and AMP substrates, where the results were represented as mean±SE, n = 3. Statistical analysis was performed by one-way ANOVA with Dunnett’s post-test for multiple comparisons. *significantly decreased compared to wild-type strain SK36 (P<0.05). Δ*nt5e*: 5′-nucleotidase deletion mutant; Δ*nucH*: extracellular nuclease deletion mutant; Δ*cnp*: cyclo-nucleotide phosphodiesterase deletion mutant; and Δ*rad3:* DNA repair ATPase deletion mutant.

By mining the SK36 genome, 16 putative surface proteins with LPxTG motifs were identified. Of these proteins, we identified four putative cell-surface nucleotidases based upon the presence of an LPxTG motif and substrate specificities that suggest the ability to hydrolyze a nucleotide and water to a nucleoside and orthophosphate (Table S1). Putative Nt5e showed a substrate specificity that would most likely hydrolyze released platelet adenine nucleotides and yield adenosine. To test this possibility in strain SK36, allelic exchange mutants of *nt5e* and the other putative nucleotidases were each constructed using primers listed in Tables S3 (SK36) and compared for loss of hydrolysis of extracellular adenine nucleotides (Figure 1). Each strain was incubated with 10, 50, or 100 µM of ATP, ADP or AMP. Hydrolysis is reported as *S. sanguinis* Nt5e activity per cell. When strain SK36 was compared to deletion mutants of genes for the extracellular nuclease (*nucH*), cyclo-nucleotide phosphodiesterase (*cnp*) or DNA repair ATPase (*rad3*), only Δ*nt5e* cells lost significant ability of whole cells to hydrolyze ATP (Figure 1A, P<0.05), ADP (Figure 1B, P<0.05) and AMP (Figure 1C, P<0.05) at each concentration examined. The ATPase, ADPase and AMPase activities of Δ*nt5e* cells were also associated with a reduction in the lag time to platelet aggregation (Figure S1).

**Table 1:** Characterization of the enzymatic activities of *S. sanguinis* 133-79 Nt5e.

Substrate	ATP		ADP		AMP	
	K_m_ *^a^*	V_max_ *^b^*	K_m_	V_max_	K_m_	V_max_
wt	118.0±35.1	9.0±1.0	65.7±20.0	5.4±0.5	38.9±15.6	1.0±0.2
Δ*nt5e*	396.0±25.9^c^	1.7±0.9^c^	–	–	–	–
*nt5e*+	124.7±33.8	8.3±0.9	81.7±21.1	5.4±0.5	40.0±7.2	0.9±0.1

ab Enzyme parameters were calculated by nonlinear curve fitting using GraphPad Prism. K_m_ was represented as mean.

±SE µM and V_max_ was represented as mean±SE nmoles Pi/min per 10^6^ cells.

c Statistical analysis was performed using ANOVA, and followed by Bonferroni test. Significant differences were only. obtained when comparing wt and Δ*nt5e,* P<0.01.

**Figure 2:**
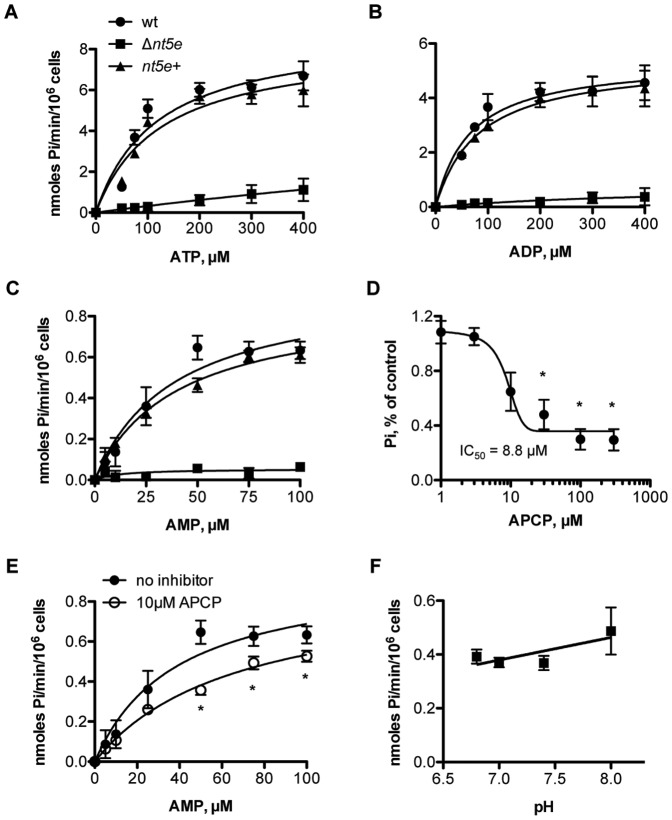
Characterization of Nt5e activity on *S. sanguinis* 133-79 whole cells. Nt5e activity was measured by the release of inorganic phosphate (Pi) from adenine nucleotides. For (A), (B), and (C), the Michaelis-Menten curves were showed as enzyme velocity (represented as nmole/min/10^6^ cells) vs. concentration of ATP, ADP and AMP substrates. (D) Effect of Nt5e inhibitor APCP on AMPase activity of *S. sanguinis* 133-79. The curve was fitted to a sigmoidal inhibitory dose-response curve and the inhibitory concentration 50% (IC_50_) value derived from the curve fit was shown. (E) Michaelis-Menten curves of AMPase activity vs. substrate concentration in the absence and presence of APCP. (F) pH dependence of AMPase activity of Nt5e. Statistical analysis was performed using non-linear regression. The results were represented as mean±SE, n = 3; *significantly decreased compared to no inhibitor (P<0.05).

### Characterization of the Enzymatic Activities of *S.* *sanguinis* 133-79 Nt5e

Platelets from some donors do not aggregate in response to strain SK36, reflecting donor specificity [21]. To determine whether the enzymatic activities were expressed by other platelet-aggregating strains, we also studied *S. sanguinis* 133-79. Whole cells of *S. sanguinis* 133-79 wild-type (wt) hydrolyzed the adenine nucleotides ATP (Figure 2A), ADP (Figure 2B) and AMP (Figure 2C), following Michaelis-Menten kinetics. The K_m_ and V_max_ for the different strains tested are summarized in Table 1. Using the Δ*nt5e* strain, the ATPase activity was significantly decreased (P<0.01), whereas activity in this mutant was restored to wt levels by complementation (*nt5e*+) (Figure 2A). In the absence of *nt5e*, the ADPase activity was reduced, whereas activity in this mutant was restored to wt levels by complementation (*nt5e*+) (Figure 2B). Similarly, AMPase activity in *S. sanguinis* 133-79 was fully abrogated by deletion of *nt5e* (Figure 2C). When complemented, *nt5e*+ regained AMPase activity. Indeed, Δ*nt5e* generated inorganic phosphate only with ATP as substrate, which may reflect activity of an ecto-ATPase on the cell surface of *S. sanguinis* as we reported [9]. When compared to NT5E, ecto-ATPase activity did not play a major role in the hydrolysis of extracellular adenine nucleotides. Collectively, these results suggest strongly that Nt5e on *S. sanguinis* 133-79 hydrolyzes the adenine nucleotides ATP, ADP and AMP.

### AMPase Activity Attributed to Nt5e

We focused on the ability of *S. sanguinis* to produce adenosine, which could potentially affect the course of experimental endocarditis. Since our assays were performed with whole cells, we considered the possibility that total enzyme activity could include other phosphatases. A search of the *S. sanguinis* genome suggested the possible expression of two putative alkaline phosphatase (AP) ectoenzymes [13]. To better define the enzymes responsible for whole cell AMPase activity, the assay was run in the presence of α,β-methylene ADP (APCP), a known inhibitor of Nt5e [22] (Figures 2D and 2E). When expressed on a logarithmic scale, the APCP dose-inhibition curve was fitted to a sigmoidal, single-site model (Figure 2D), allowing determination of the half-maximal inhibitory concentration (IC_50_ = 8.8±3.8 µM). In the presence of APCP (K_m_ = 66.7±14.1 µM; V_max_ = 0.9±0.1 nmoles Pi/min per 10^6^ cells), the whole cell AMPase activity showed an apparent increase in K_m_ (P<0.05), but no increase in the V_max_ (P = 0.3) compared to the no inhibitor control (K_m_ = 38.9±15.6 µM; V_max_ = 1.0±0.2 nmoles Pi/min per 10^6^ cells). Since the inhibition could be overcome at high concentrations of AMP substrate, APCP competitively inhibited AMPase activity (Figure 2E). Furthermore unlike Nt5e, AP is sensitive to an alkaline pH optimum [23]. In *S. sanguinis* 133-79, the AMPase activity did not significantly increase with increasing pH (Figure 2F). Therefore, the AMPase activity was not AP, but attributable to Nt5e.

### Identification of Nt5e as a Cell-surface Protein from *S.* *sanguinis*

**Figure 3:**
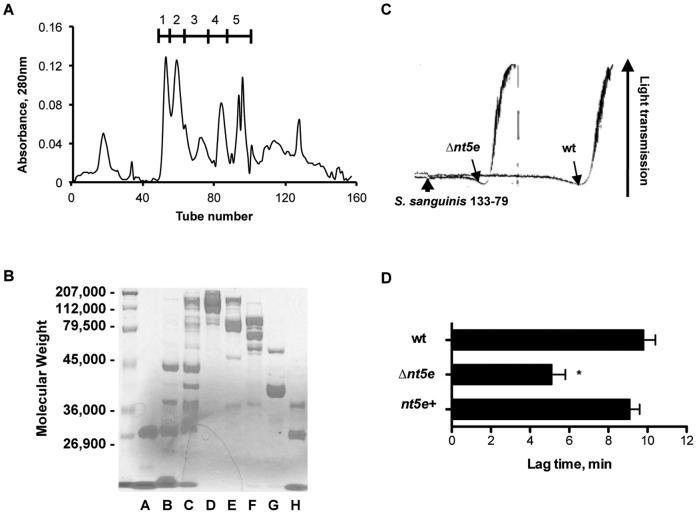
Nt5e is a trypsin-cleavable surface protein of *S. sanguinis* and affects platelet aggregation lag time. (A) Gel filtration chromatography of 7-minute tryptic digest of *S. sanguinis* 133-79. 6.4****mg was placed on a column of Sephadex G-100 and chromatographed as described under “Materials and methods”. (B) SDS-PAGE analysis of *S. sanguinis* tryptic digest fractions from gel filtration chromatography. All samples contained 15 µg of protein solubilized in 1% (w/v) SDS sample buffer. These samples were electrophoresed on a 10% gel, and stained with Coomassie Blue. Lane A, trypsin. Lane B, flow-through from void volume. Lane C, starting 7-minute crude tryptic digest. Lane D, Sephadex G-100 fraction 1. Lane E, Sephadex G-100 fraction 2. Lane F, Sephadex G-100 fraction 3. Lane G, Sephadex G-100 fraction 4. Lane H, Sephadex G-100 fraction 5. (C) PRP was stirred in an aggregometer. Wild type and Δ*nt5e* strains were added at the *S. sanguinis* 133-79-labeled arrowhead and aggregation was measured as increasing light transmission. The start of aggregation of each strain was indicated by arrow. The aggregation tracing in response to the *nt5e*+ strain (not shown) was indistinguishable from the wild type. (D) Response leading to aggregation was recorded as the mean lag-time to onset of aggregation±SE, N = 4; Statistical analysis was performed using one-way ANOVA with Tukey-Kramer post-test for multiple comparisons. * significantly decreased compared to wt (P<0.05).

To show that Nt5e is a cell wall-associated enzyme, we characterized a cell surface protein fraction with the requisite enzyme activities. Recovery of the cell surface proteins fragments from *S. sanguinis* 133-79 was maximal after 7 minutes of TPCK-trypsin digestion (data not shown). The 7-minute tryptic digests (crude digest) of *S. sanguinis* 133-79 were chromatographed on a column of Sephadex G-100, pooled (Figure 3A), fractions resolved using SDS-PAGE (Figure 3B), and analyzed for platelet interactions (Table 2). Fraction 3 (G100-3) had the greatest ability to inhibit *S. sanguinis*-induced platelet aggregation, but had no effect on platelet-*S. sanguinis* adhesion (data not shown). After separation of Fraction 3 by two-dimensional SDS gel electrophoresis and analysis by mass spectrometry, two putative 5′-nucleotidase superfamily proteins were identified (Table 3) [13]. The crude digest and fraction G100-3 showed 5′-nucleotidase activities (Table 2). Compared with the crude digest, G100-3 had higher ATPase, ADPase and AMPase activities, which correlated with the inhibition of platelet aggregation (Table 2). In the plasma, both ADP removal and adenosine generation can inhibit platelet aggregation [24]. Taken together, 5′-nucleotidase is a protein cleaved from the surface of *S. sanguinis* 133-79, which appeared to metabolize adenine nucleotides to modulate platelet aggregation.

**Table 2:** Recovery of Sephadex G100 fractions of S. sanguinis 133-79 tryptic digest.

			Hydrolysis, nmoles Pi/ng/min^b^		
Fraction	Protein, mg(recovered %)	PRP aggregationonset, min^a^	AMP	ADP	ATP
None	–	7.5	–	–	–
Crude digest	6.4	9.8	0.15	0.12	0.11
Fraction 1	1.3 (20.3)	7.5	0.01	0.01	0.01
Fraction 2	1.7 (26.6)	7.8	0.12	0.10	0.09
Fraction 3	1.0 (15.6)	>30	0.59	0.55	0.42
Fraction 4	0.8 (12.5)	7.6	0.01	0.01	0
Fraction 5	0.8 (12.5)	7.5	0.02	0	0
Recovered total	5.6 (87.5)				

a PRP was preincubated with the indicated fraction at a final concentration of 0.1 mg/ml.

b Proteins were incubated with 50 µM of AMP, ADP or ATP for 15 minutes at 37°C at a final concentration of 10 µg/ml.

**Table 3:** Proteins in G100-3 identified by mass spectrometry.

Identified protein	Accession No.	ID Probability	Putative substrate specificity
5′-nucleotidase, putative (*S. sanguinis* SK36)	gi|125718054	95%	5′-nucleotides with preference for adenine nucleotides
Cyclo-nucleotide phosphodiesterase (*S. sanguinis* SK36)	gi|125717119	100%	Nucleoside 2′,3′-cyclic phosphate into nucleoside 3′-phosphate
Cyclo-nucleotide phosphodiesterase (*S. suis* 98HAH33)	gi|146321945	100%	Nucleoside 2′,3′-cyclic phosphate into nucleoside 3′-phosphate
5′-nucleotidase family protein (*S. gordonii* CH1)	gi|157150885	99%	5′-nucleotides with preference for adenine nucleotides

### Inhibition of Platelet Aggregation by Nt5e

We next compared whole cells of *S. sanguinis* 133-79 wt, Δ*nt5e* and *nt5e*+ strains for the ability to induce platelet aggregation. In response to either wt or *nt5e*+, platelets from a representative donor aggregated in approximately 9 minutes (Figure 3C). The aggregation response of Δ*nt5e* showed a lag-time of approximately 5 minutes, which was significantly shorter than the wt and *nt5e*+ strains (Figure 3D). The magnitude of aggregation, however, was similar in response to all three strains (Figure 3C). The aggregation responses to *S. sanguinis* 133-79 wt, Δ*nt5e* and *nt5e*+ were consistent for all three platelet donors tested (data not shown). Similar results were obtained using *S. sanguinis* SK36 (Figure S1). Therefore, *S.* *sanguinis* Nt5e prolonged the lag time to the onset of platelet aggregation.

### Adhesion of Platelets and Bacteria

Since the ability to induce aggregation is a function of adhesion of *S. sanguinis* to platelets [25], we next sought to determine whether deletion of NT5E affected aggregation because adhesion was affected. The percent adhesion to platelets was similar for wt (133-79: 60.1±0.9%; SK36: 60.1±1.9%), Δ*nt5e* (133-79: 59.9±1.2%; SK36: 56.5±1.4%) and *nt5e+* (133-79: 59.6±2.6%) strains. Hence, NT5E modulates platelet aggregation without affecting adhesion.

### Nt5e Contributes to the Virulence of *S. sanguinis* in Rabbit Infective Endocarditis

**Figure 4:**
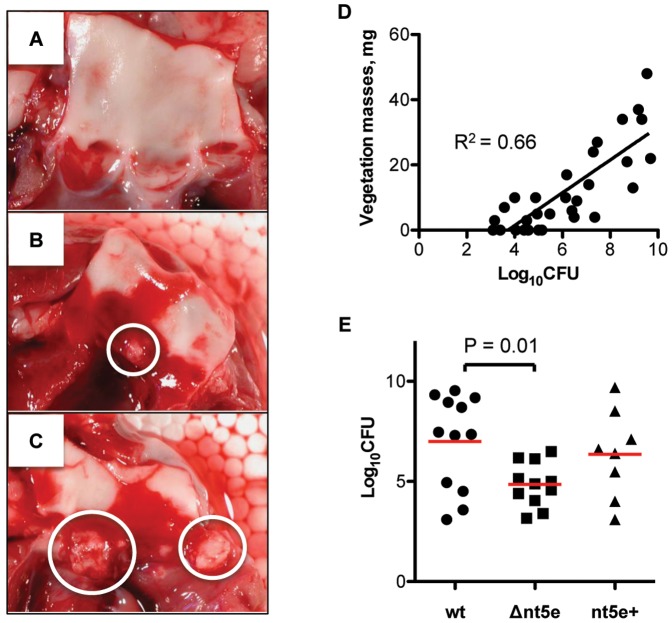
Nt5e affects vegetation weight and bacterial load in a *S. sanguinis* rabbit endocarditis model. (A) Aortic valve of a rabbit infected with *S.* *sanguinis* 133-79 Δ*nt5e*. The aortic valve is composed of three leaflets, no visible vegetations were found and 2.5×10^3^ CFUs were recovered. (B) Aortic valve of a rabbit infected with *S. sanguinis* 133-79 *nt5e*+, with one vegetation in the center leaflet (white circle) and 3.3×10^8^ CFUs were recovered. (C) Aortic valve of a rabbit infected with *S. sanguinis* 133-79 wt, with two vegetations on center and right leaflets (white circle), respectively and 3.4×10^9^ CFUs were recovered. (D) Plot of vegetation bacterial load (total CFU) versus vegetation mass. All vegetations on the aortic valve of each rabbit were pooled to obtain the vegetation weight and bacterial load (on TH plate). When no vegetations were found, the valves were scraped with a blade and plated to determine the valve bacterial load. R^2^ = 0.66 (n = 31) indicated that there is a correlation between the bacterial load and vegetation masses. (E) Bacterial loads in the rabbit endocarditis model, enumerated as log_10_ total CFU 4 days after infection. Statistical analysis was performed using one-way ANOVA with Tukey-Kramer post-test. Horizontal bars represent mean CFUs in each cohort.

To investigate the contribution of Nt5e to *S. sanguinis* virulence in infective endocarditis, rabbits with experimental heart valve injury were infected by intravenous inoculation with 1×10^9^ CFU *S. sanguinis* 133-79 wt, Δ*nt5e*, or *nt5e*+. Growth curves in TH broth were performed for all three strains to test for changes in cell growth or fitness. Although the *nt5e* deletion mutant showed a slight delay in entry to log-phase, all strains displayed similar growth rates in TH broth maintained in air with 5% CO_2_ (not shown). The resulting vegetations ranged from non-apparent (Figure 4A) to macroscopic lesions (Figures 4B and 4C).

Four days after inoculation with *S. sanguinis*, bacterial load recovered from the vegetations ranged from 10^3^ to 10^9^ per rabbit; the vegetative masses correlated with the recovered bacterial CFUs (Figure 4D; R^2^ = 0.66, n = 31). Eight of 31 infected rabbits had no visible vegetations, which might due to insignificant injury caused by catheter placement. Total recovered bacterial CFUs indicated the relative ability of each strain to colonize and proliferate on the heart valve.

As expected, 11 out of 12 rabbits infected with wt formed aortic vegetations with a mean mass of 18.6 mg and mean recovered bacterial load of 1.0×10^8^ CFU. Similarly, after infection with *nt5e*+, vegetations formed in 7 of 8 rabbits with a mean mass of 12.5 mg and mean bacterial load of 0.2×10^8^ CFU on TH plates, and 0.3×10^6^ CFU on TH plates with appropriate antibiotics. The CFUs recovered with and without antibiotics were not significantly different. In contrast, 6 of 11 rabbits injected with Δ*nt5e* showed no vegetations. Two other rabbits died before euthanasia and were excluded from statistical analysis. In rabbits challenged with Δ*nt5e*, the mean weight of vegetations was 4.0 mg and the mean bacterial load was 0.7×10^6^ CFU on non-selective and 0.1×10^6^ CFU on selective plates, which were the lowest infectious loads among these three groups. Taken together, Δ*nt5e*-infected rabbits developed significantly smaller aortic vegetations (P<0.01) with significantly lower bacterial CFUs (P = 0.01) than rabbits challenged with the wt strain. Notably, the Δ*nt5e* group showed an approximately 100-fold reduction in mean CFUs as compared with the wt group (Figure 4E). After rescuing the *nt5e* gene, *nt5e*+ and wt infected rabbits showed comparable aortic vegetations (P = 0.32) and bacterial CFUs (P = 0.55).

## Discussion

For the first time, we present data that strongly suggests *S. sanguinis* Nt5e contributes to virulence during experimental IE in rabbits. This cell surface enzyme can hydrolyze ATP to adenosine, which slows the platelet aggregation response in vitro and may reduce the accumulation of platelets on infected heart valves in vivo. As a surface protein, Nt5e could also be involved in adherence to platelets or platelet vegetations and contribute to nutrient acquisition (i.e., nucleosides) and the persistence of infection. Other than adenosine production, these explanations appear unlikely. In the presence and absence of Nt5e, adhesion of *S. sanguinis* to platelets is similar as we show, and growth and biofilm formation were also comparable (data not shown). It is more plausible that during IE adenosine is produced to inhibit further platelet aggregation and also function as an immunosuppressive agent. In this context, immunosuppressive adenosine has recently been implicated in other infections caused by bacteria including *Staphylococcus aureus*, *Bacillus anthracis* and also the protozoan parasite *Leishmania* [26]–[28]. Since Nt5e is widely conserved, the hydrolysis of ATP to immunosuppressive adenosine in vivo may be relevant to the virulence of infection by many species. Direct evidence for the role of adenosine produced by Nt5e during infection is needed.

As a potent immunoregulator of inflammation, adenosine inhibits the proinflammatory and promotes anti-inflammatory actions of immune cells via four specific cell surface receptors: A_1_, A_2a_, A_2b_, and A_3_ [29]. For example, in human and mouse monocytes/macrophages, the engagement of adenosine receptors, particularly A_2a_ receptors, by adenosine or its analogues inhibits the production of IL-12 and TNF-α, two proinflammatory cytokines, and increases IL-10, a protective cytokine that suppresses IL-12 and TNF-α [19], [20], [30]–[32].

To understand the mechanism by which adenosine is produced, we began with our earlier observation that nucleotidase activity is expressed on the cell wall of *S. sanguinis* [9], [10]. The fact that the Nt5e deletion mutant possesses ATPase activity at higher ATP concentrations suggests a contributory role of the ecto-ATPase (higher K_m_) in the hydrolysis of ATP. In the present report, we distinguish the ecto-ATPase activity from Nt5e. We characterized nucleotidase activities associated with whole cells and showed that adenosine was the product of Nt5e hydrolysis of ATP, ADP or AMP in vitro. For each substrate, hydrolysis by whole cells followed Michaelis-Menten kinetics and could not be attributable to other enzyme activities associated with phosphate hydrolysis. These results and analysis of the published genome of strain SK36 and our partially sequenced strain 133-79, led us to hypothesize that the surface nucleotidase activities were attributable to a single surface enzyme, Nt5e on *S. sanguinis*.

To explore the possibility that adenosine-producing activity could resolve to a single protein, Nt5e was cleaved from the cell surface of *S. sanguinis* 133-79 by mild trypsin digestion. When the digest was fractionated using gel filtration chromatography, Nt5e largely resolved in one of the fractions, G100-3, and its presence was confirmed by mass spectroscopic analysis. The possibility that other enzymes detected in this fraction could mimic Nt5e activity was systematically excluded. Since Nt5e can hydrolyze ATP and ADP, an agonist of platelet aggregation, and generate adenosine, an inhibitor of platelet aggregation, it was not surprising that fraction G100-3 inhibited platelet aggregation. Nt5e contains an LPxTG-motif, which is characteristic of cell wall bound surface proteins in Gram-positive bacteria [14]. Given that enzyme activity is also expressed on intact, viable whole cells of *S. sanguinis*, Nt5e is an ecto-enzyme. Since the Δ*nt5e* strain was unable to hydrolyze the nucleotides to adenosine, the data strongly suggest that cell-surface Nt5e activity modulates platelet aggregation by producing adenosine from related nucleotides.

In the absence of *nt5e*, the ATP and ADP released from platelet dense granules are not hydrolyzed, adenosine is not produced and the lag time to the onset of platelet aggregation is shortened. We largely ruled out that the loss of surface Δ*nt5e* affected interactions with the platelet surface since this mutant showed platelet adhesion similar to the wild-type strain. In the interactive microenvironment with platelets, *S. sanguinis* induces release of ATP and ADP from platelet dense granules [9], [10]. *S. sanguinis* Nt5e is, therefore, strongly suggested to hydrolyze ATP and ADP released from dense granules and produce aggregation inhibitor adenosine to modulate aggregation responses.

In IE, the ability of *S. sanguinis* to induce platelet aggregation in vitro is directly related to the mass of developing thrombotic vegetations on heart valves in vivo [4]–[6], [33]. Using a rabbit endocarditis model, we compared the virulence of *S. sanguinis* 133-79 wt, Δ*nt5e*, and *nt5e*+ in vivo. Virulence was reflected by the weight of resulting cardiac vegetations and bacterial burden [4], [34]. The mass of the vegetations was directly related to the CFUs of bacteria colonizing the vegetations (R^2^ = 0.66, n = 31) when total CFUs of all strains were enumerated on non-antibiotic-containing plates. In Δ*nt5e* challenged rabbits, the mass of the vegetations and recovered bacterial CFUs were 100-fold lower than in wt infected rabbits. When complemented, *nt5e*+ and wt infected rabbits showed comparable aortic vegetations and bacterial CFUs on plates with no antibiotics. Based on these data, *nt5e* appears to be responsible for the increased virulence of the wild-type and complemented strains.

Differences in vegetation formation and recovery of CFUs from the heart valves of infected rabbits may reflect strain-specific efficiency in bacterial clearance from heart valves. When Nt5e was expressed by infecting wt and *nt5e*+ strains, adenosine produced in the vegetation microenvironment could inhibit phagocytosis by infiltrating monocytes/macrophages to facilitate colonization [35]. The concentrations of locally available adenosine would be higher than in vegetations from the Δ*nt5e* group, which showed attenuated vegetation formation and lower recovered CFUs. In addition, wt and *nt5e*+ strains would hydrolyze ATP in the microenvironment. Extracellular ATP, a proinflammatory signal, can be sensed by purinergic P2Y receptors, which are highly expressed on immune and non-immune cells [36]. On endothelial cells and monocytes, stimulation with extracellular ATP leads to the release of proinflammatory cytokines such as IL-1β and IL-12 [18], [20], [37]. When ATP is hydrolyzed and adenosine is generated, proinflammatory activity would be expected to be lost and immunosuppressive activity produced. Therefore, Nt5e potentially promotes bacterial survival through ATP removal and adenosine generation. How *nt5e* might contribute, however, to bacteria-monocyte interactions remains to be established. We await further direct evidence for the role of Nt5e nucleotidase activities in IE; a catalytic mutant of Nt5e will be informative when developed.

Nt5e could also play another role in the formation of vegetations in vivo. Both rabbit [38] and human platelets [39] release platelet microbicidal proteins (PMPs) and thrombin-inducible PMPs (tPMPs) in vitro. The PMPs and tPMPs can both kill [40] and exert nonlethal anti-adherence effects [41] against a wide spectrum of endovascular pathogens, including viridans streptococci [40]. Since the Δ*nt5e* strain induced platelet aggregation faster than *S. sanguinis* isogenic wild-type strain, without affecting bacterial-platelet adhesion, release of PMPs would also be affected. In IE, the Δ*nt5e* strain might thwart early infection more efficiently than when *nt5e* is expressed. With less time for growth and doubling of the infecting bacteria, the mass of the vegetation and eventual bacterial load will be lower than in the presence of NT5E. Using two plausible mechanisms, therefore, Nt5e may contribute to the bacterial survival in the blood, persistence in the vegetation, and virulence of the organism.

In a rabbit IE model, Turner et al. [12] reported that a *nt5e* transposon mutant of *S. sanguinis* SK36 and the parental strain were equally competitive. These findings differed from our own. The different findings may reflect the study designs, including implementation of the animal model, the difference in the construction of the mutant, the inoculum size, and the duration of infection.

Of note also, some rabbits developed lung congestion. During IE, formation of large aggregates of bacteria and platelets would be expected to obstruct small capillaries, such as those in the lungs [4], [33]. However, lung congestion did not appear to be associated with *nt5e* or size of vegetation (data not shown).

Note also that the bacterial counts from rabbits infected with Δ*nt5e* were not significantly different, based on CFUs recovered from antibiotic-containing and non-antibiotic plates. Plating efficiency was similar. For rabbits infected with *nt5e*+, less than 1% of the post-infection bacteria enumerated from the vegetations appeared to retain the complemented plasmid, indicating antibiotic resistance tended to be cured without the selective pressure of the specific antibiotics. The vegetation masses and bacterial CFUs recovered from non-antibiotic plates of *nt5e*+ and wt groups were comparable, however, suggesting that *nt5e*+ loses the complemented plasmid gradually during the course of infection. These results suggest that the impact of Nt5e on the persistence of *S. sanguinis* in the infected vegetation may be underestimated by our studies.

In summary, we have shown that Nt5e possesses specific nucleotidase activity, which can influence platelet aggregation by the production of adenosine. Adenosine is likely to influence the outcome of *Streptococcus sanguinis* infection. To show that specific nucleotidase activity of Nt5e is a contributory factor towards the development of infective endocarditis, specific enzymatic activity must be mutated within the surface enzyme. In our animal model of endocarditis, we did show that Nt5e deletion attenuates virulence. We cannot completely exclude the possibility, however, that Nt5e may possess other properties that contribute towards this phenotype. Cell-surface Nt5e activities are widely distributed among bacteria including *Staphylococcus aureus* [42], *Helicobacter pylori* [43] and *S. gordonii* [44]. The platelet modulating effects of Nt5e might be a common mechanism among *S. sanguinis* strains, and perhaps other bacterial pathogens, such as staphylococci and enterococci, which cause blood borne infections. Indeed, AMPases on pathogens like *Staphylococcus epidermidis* and *Enterococcus faecilis* can generate immunosuppressive adenosine [28]. Therefore, the nucleotidase activity of Nt5e is suggested to be a common virulence factor for the survival of bacterial pathogens in the blood.

## Materials and Methods

### Bacterial Strains and Culture Conditions

*S. sanguinis* strains were routinely grown in Todd Hewitt broth (TH broth, Difco; Sparks, MD) or on TH agar plates at 37°C in 5% CO_2_. *E. coli* cells were grown aerobically at 37°C in Luria-Bertani broth (LB broth, Bacto; Sparks, MD). When required, antibiotics were added to the medium at the indicated concentrations: erythromycin (Em), 10 µg ml^−1^ (*S. sanguinis*); and kanamycin (Km), 50 µg ml^−1^ (*E. coli*) or 400 µg ml^−1^ (*S. sanguinis*).

### Genetic Manipulations in *S. sanguinis*

Standard recombinant DNA techniques were employed as described [43]. Plasmids (listed in Table S2) were purified from *E.* *coli* cells using the QIAquick Spin Miniprep purification Kit (Qiagen Inc., Valencia, CA). Oligonucleotides for strain SK36 (Table S3) and 133-79 (Table S4) were synthesized for deletion (A1 and A2) and complementation (ACom) using integrated DNA Technologies.

Chromosomal DNA was prepared from mutanolysin-treated streptococcal cells using the Qiagen 100/G Genomic Tip system [45]. PCR products were purified using the High Pure PCR Product Purification Kit (Roche Ltd., Indianapolis, IN). DNA restriction and modification enzymes were used according to the manufacturer’s directions (New England Biolabs Inc., Ipswich, MA).

The target genes of *S. sanguinis* (wt) were inactivated by allelic exchange with the erythromycin-resistance determinant, *ermAM*. Briefly, *ermAM* was amplified from plasmid pVA891 [46] and cloned into pPCR-Amp SK (+) (Stratagene Corp., La Jolla, CA). Two DNA fragments constituting the flanking sequences of the target genes were then amplified and fused with the *ermAM* genes sequentially [47]. The fused construct was then PCR-amplified, purified and transformed into wt. For transformation, overnight cultures were grown in TH broth. The next day, cells were inoculated into fresh aliquots of the same medium (1∶40), containing 10% heat-inactivated horse serum (Sigma-Aldrich, St. Louis, MO), and the fused DNA construct was added. Competence stimulating peptide (CSP, a gift from Dr. Jens Kreth, University of Oklahoma) was added to a final concentration of 200 ng ml^−1^. Incubation continued for 5 h at 37°C and cells were plated on Em selective TH plates, generating the deletion mutant. The mutation was confirmed by PCR amplification and sequencing.

To complement the deletion mutant, a DNA fragment encompassing the entire target gene and upstream promoter sequence was amplified by PCR from wt and cloned into the *E.* *coli*-streptococcal shuttle vector pDL276 [48], generating plasmid pDL276-gene. The construct was amplified in *E. coli*, purified and used to transform the deletion mutant to obtain the complemented strain using the method described above.

### Nucleotidase Activities

Streptococcal cells in stationary phase were harvested from overnight cultures. Harvested cells were washed twice with 30 mM Tris•HCl buffer (pH 7.4) containing 0.25 mM ethylenediaminetetraacetic acid (EDTA) and 30 mM sodium chloride (NaCl), followed by washing with 50 mM Tris•HCl (pH 7.4) containing 130 mM sodium chloride (NaCl) and 5 mM magnesium dichloride (MgCl_2_), and resuspended to 2×10^9^ cells per ml. For phosphate hydrolase activity, streptococcal cells were washed twice with 50 mM Tris•HCl (pH 8.0) buffer containing 150 mM NaCl, 5 mM CaCl_2_, and 5 mM MgCl_2_. The bacterial suspension (0.5 ml) was mixed with AMP at final concentrations up to 100 µM in a 2 ml microcentrifuge tube, or with up to 400 µl ADP or ATP, and incubated at 37°C for 30 minutes. After incubation, cells were centrifuged at 10,000×g for 5 minutes, and 50 µl of the supernatant was transferred into 96-well plates (Corning, N.Y.). Similarly, the crude tryptic digest or fractions from gel filtration (final concentration 10 µg/ml) were incubated with 50 µM AMP, ADP or ATP at 37°C for 15 minutes. The reactions were stopped with an equal volume of HCl (final concentration 0.1N). The final solution (50 µl) was then transferred into 96-well plates. The enzymatic activity was measured as the amount of inorganic phosphate (Pi) released into the supernatants using the QuantiChrom Phosphate Assay Kit DIPI-500 (Bioassay systems, Hayward, CA). The results were expressed as nM of Pi produced/min per 10^6^ cells or nM of Pi produced/min per ng of protein. The K_m_ (Michaelis constant) and V_max_ (maximum velocity) for AMPase activity of intact *S. sanguinis* cells were calculated from substrate concentration curves using nonlinear regression of four replicates for each concentration point. To minimize the likelihood that Pi was generated by other enzymes, some streptococcal cells were pretreated with 1 µM to 1 mM tetramisole (Sigma-Aldrich, St. Louis, MO), an inhibitor of alkaline phosphatase, or 1 µM to 1 mM adenosine 5′-[α,β-methylene] diphosphate (APCP) (Sigma-Aldrich, St. Louis, MO), an inhibitor of mammalian Nt5e, before incubating with AMP.

### Biochemical Characterization of NT5E in Cell Wall Fractions

Minimal tryptic digests of *S. sanguinis* 133-79 were prepared as described [49], which leaves the cell wall intact. The crude digests representing minimally digested surface proteins were concentrated and desalted using an ultrafiltration column (10 kDa cutoff; Millipore, Billerica, MA) into 2 mL deionized water (dH_2_O). The salt-free protein fragment concentrates were then chromatographed on a column (1.25×95 cm) of Sephadex G-100 (GE Healthcare, Pittsburgh, PA) at a flow rate of 0.3 ml/min in PBS. The fraction with the greatest ability to inhibit *S. sanguinis*-induced platelet-rich plasma (PRP) aggregation (G100-3) was then analyzed using two-dimensional SDS gel electrophoresis. Gels were stained with silver stain and spots were excised for mass spectrometry analysis (Center for Mass Spectrometry and Proteomics, University of Minnesota).

### Platelet Aggregometry

Strains of *S. sanguinis* were tested for the ability to induce platelet aggregation using fresh PRP obtained from a single donor as described previously [24]. A single donor was used to eliminate variability in platelet response between donors [21] and the procedures were reviewed and approved by the IRB of the University of Minnesota. Each bacterial strain (50 µl suspension containing 4×10^9^ cells/ml) was incubated with 450 µl of PRP (4×10^8^ cells/ml). PRP aggregation was performed at 37°C with controlled stirring in a recording aggregometer (model 660, Chronolog Corp., Havertown, PA), and the lag time or delay to onset (minutes) was measured.

### Platelet Bacterial Adhesion Assay (PBAA)

All procedures were performed as described previously [24]. In brief, platelets from outdated PRP (Memorial Blood Center, St. Paul, MN) were washed with PBS and fixed with 10% formalin. Washed platelets and washed streptococcal cells were incubated together or alone (controls) in microwells; the small clusters of adhering platelets and bacteria were separated from non-interacting particles by centrifugation. The sedimentation of adhering mixtures relative to controls was quantitated by the following formula: percent adhesion = 100×{1–[mixture *A_620 nm_*/(bacterium *A_620 nm_*+washed-platelet *A_620 nm_*)/2]}. Based on previous studies of the variability of the method, only adhesion scores of ≥20% were considered positive.

### Experimental Endocarditis Model

Injury-induced experimental endocarditis was initiated by placement of a catheter into the left side of the heart in healthy, adult New Zealand White rabbits (2 to 3 kg; obtained from Bakkom Rabbitry, Red Wing, MN), essentially as described previously [4], [34]. The procedures were reviewed and approved by the IACUC of the University of Minnesota. The catheter was retained in place for 2 hours to abrade the heart valve and then removed. After closure of the neck incision, viable *S. sanguinis* was injected intravenously via the marginal ear vein. A total of 33 rabbits were inoculated with 1×10^9^ of *S. sanguinis* 133-79 wt (n = 12 rabbits), *nt5e* deletion mutant (Δ*nt5e*) (n = 13) and complemented strain (*nt5e*+) (n = 8). After four days, the animals were euthanized, hearts were removed, aortic valves were excised and vegetations were weighed. To determine infecting bacterial colony forming units (CFUs), vegetations from each rabbit were homogenized separately in 1 ml of TH broth and plated onto TH agar and CFUs were enumerated.

To determine whether antibiotic resistance markers were lost during the four-day infection in vivo without antibiotics, samples from vegetations were also enumerated on replica plates with appropriate antibiotics. For Δ*nt5e*, the CFUs on TH plates with or without antibiotics were not significantly different (P = 0.25), indicating that resistance markers were retained. In contrast, the *nt5e*+ group tended to show reduced growth on antibiotic-containing medium (P = 0.15), suggesting loss of the complementation plasmid. Less than 1% of the bacteria enumerated from the *nt5e*+ group still retained the complemented plasmid.

When vegetations were not visualized, all of the aortic valve leaflets were scraped and cultured to enumerate the bacteria colonizing the valves. Since all bacteria recovered from the aortic valve potentially contributed to the infection and vegetation formation, CFUs on TH plates without antibiotics were used for statistical analysis. All experiments were conducted under the established guidelines of the University of Minnesota Institutional Animal Care and Use Committee.

### Gene Sequences

We have partially sequenced the genome of *S. sanguinis* 133-79. The putative *nt5e* gene of *S. sanguinis* 133-79 and *S. sanguinis* SK36 [13] shared 95% sequence identity. The annotated genome of *S. sanguinis* SK36 (NC_009009) is available at http://www.ncbi.nlm.nih.gov. The GenBank accession number for the *S. sanguinis* 133-79 *nt5e* gene is BankIt1529021 Seq1 JQ920433.

### Statistical Analysis

Descriptive statistics, including the means and standard errors, were calculated. Total CFUs were converted to log_10_ values prior to statistical analysis. Statistical analysis of data was performed using the Student’s *t*-test, one-way analysis of variance (ANOVA), non-linear regression or 4-parameter logistic regression (4-PL) with GraphPad Prism 5 (GraphPad Software, La Jolla, CA). An α = 0.05 was considered to be statistically significant.

## Supporting Information

Figure S1 **NT5E affects platelet aggregation lag time in response to** ***S. sanguinis*** **SK36**. Response leading to aggregation was recorded as the mean lag-time to onset of aggregation±SD, N = 2.
(TIF)

Table S1 ***S. sanguinis*** **SK36 cell-surface proteins potentially possess nucleotidase activities.** *^a^*Available from GenBank.
(DOC)

Table S2 **Bacterial strains and plasmids used in this study** [**46]**, [48], [50], [51] **.**
(DOC)

Table S3 **Primers used in** ***S. sanguinis*** **SK36.** *^a^*All primers were designed as part of this study. *^b^*Underlined letters indicate restriction enzyme site.
(DOC)

Table S4 **Primers used in** ***S. sanguinis*** **133-79.** *^a^*All primers were designed as part of this study. *^b^*Underlined letters indicate restriction enzyme site.
(DOC)
