# Supplementary figures and images for: Ecto-5′-Nucleotidase: A Candidate Virulence Factor in Streptococcus sanguinis Experimental Endocarditis
Source: PLoS One. 2012 Jun 7;7(6):e38059. doi: 10.1371/journal.pone.0038059 (PMC3369921; doi:10.1371/journal.pone.0038059)

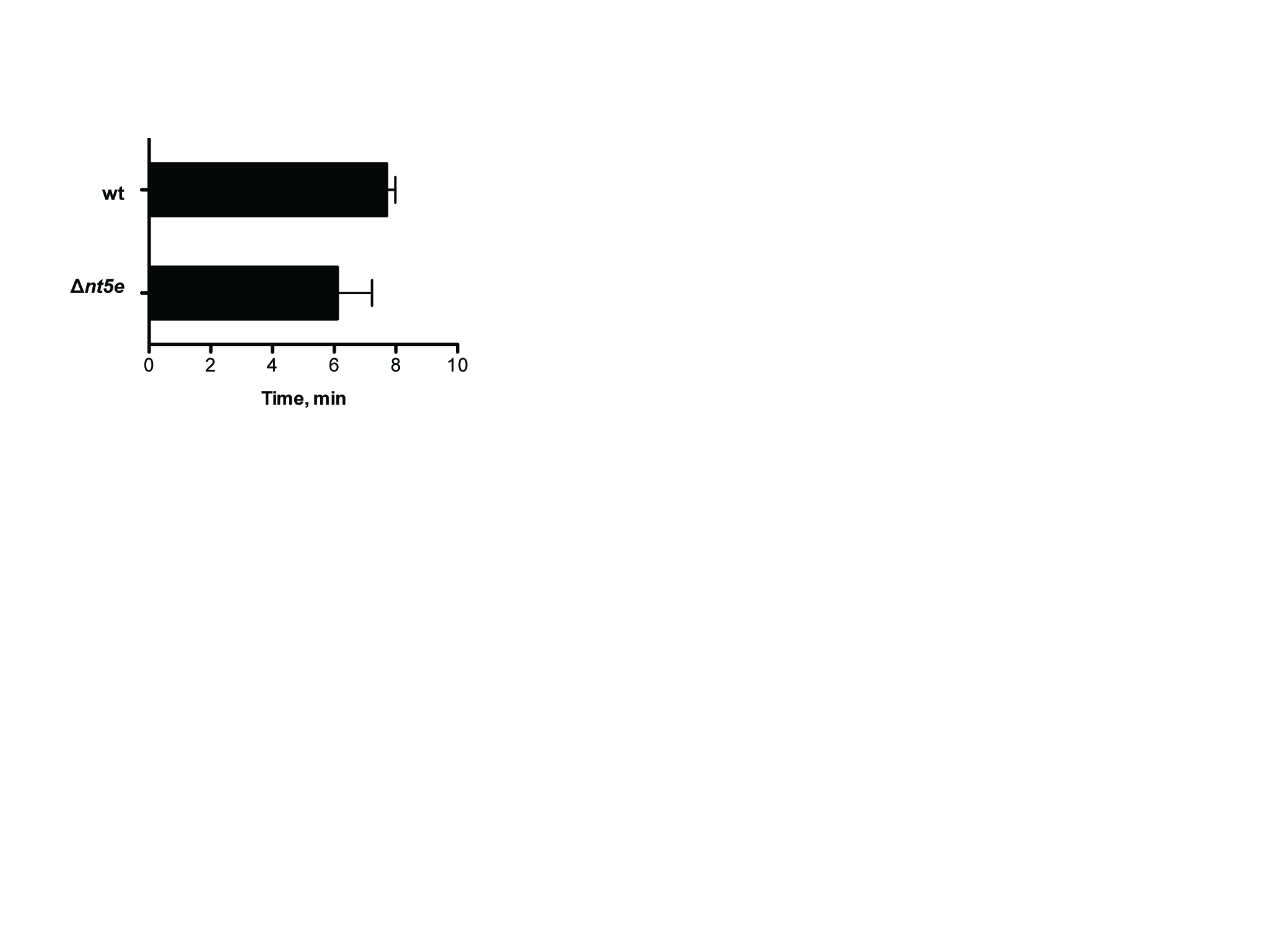

Supplement: Figure S1 — NT5E affects platelet aggregation lag time in response to S. sanguinis SK36. Response leading to aggregation was recorded as the mean lag-time to onset of aggregation±SD, N = 2. (TIF) [file pone.0038059.s001.tif]
