# Supplementary material for: Ecto-5′-Nucleotidase: A Candidate Virulence Factor in Streptococcus sanguinis Experimental Endocarditis
Source: PLoS One. 2012 Jun 7;7(6):e38059. doi: 10.1371/journal.pone.0038059 (PMC3369921; doi:10.1371/journal.pone.0038059)
Supplement: Table S1 — S. sanguinis SK36 cell-surface proteins potentially possess nucleotidase activities. aAvailable from GenBank. (DOC) [file pone.0038059.s002.doc]

**Table S1. *S. sanguinis* SK36 cell-surface proteins potentially possess nucleotidase activities**

| **Proteins, putative and genes** | **Accession No.*^a^*** | **Putative substrate specificity** |
| --- | --- | --- |
| 5'-nucleotidase, *nt5e* | SSA_1234 | 5'-nucleotides with preference for adenine nucleotides |
| extracellular nuclease, *nucH* | SSA_1750 | Ribonucleic acids to nucleoside monophosphates or diphosphates |
| cyclo-nucleotide phosphodiesterase, *cnp* | SSA_0243 | Nucleoside 2',3'-cyclic phosphate to nucleoside 3'-phosphate |
| DNA repair ATPase, *rad3* | SSA_0146 | adenosine triphosphate to adenosine diphosphate |

*^a^*Available from GenBank.
