# Supplementary material for: Ecto-5′-Nucleotidase: A Candidate Virulence Factor in Streptococcus sanguinis Experimental Endocarditis
Source: PLoS One. 2012 Jun 7;7(6):e38059. doi: 10.1371/journal.pone.0038059 (PMC3369921; doi:10.1371/journal.pone.0038059)
Supplement: Table S2 — Bacterial strains and plasmids used in this study [46] , [48], [50], [51] . (DOC) [file pone.0038059.s003.doc]

**Table S2. Bacterial strains and plasmids used in this study**

| **Strain or plasmid** | **Relevant characteristics** | **Source or reference** |
| --- | --- | --- |
| *E. coli* strain DH5α  F'I^q^ | F' *proA^+^B^+^ lacl^q^* ∆*(lacZ)M15 zzf*::Tn10(Tet^R^)/fhuA2∆(*argF-lacZ*)*U169 phoA glnV44 φ80* ∆(*lacZ*)*M15 gyrA96 recA1 relA1 endA1 thi-1 hsdR17* | New England Biolabs |
| *S. sanguinis* strains |  |  |
| 133-79 | Wild-type | [50] |
| SK36 | Wild-type | [51] |
| Plasmids |  |  |
| pPCR-Amp SK(+) | 3.0 kb; Ap^R^; pU*Cori* | Stratagene |
| pDL276 | 6.9 kb; Km^R^; ColE1*ori*, *E. coli*–streptococcal shuttle vector | [48] |
| pVA891 | 5.4 kb; Em^R^, Cm^R^; pACY*Cori*, *E. coli*–streptococcal shuttle vector | [46] |
