# Supplementary material for: Ecto-5′-Nucleotidase: A Candidate Virulence Factor in Streptococcus sanguinis Experimental Endocarditis
Source: PLoS One. 2012 Jun 7;7(6):e38059. doi: 10.1371/journal.pone.0038059 (PMC3369921; doi:10.1371/journal.pone.0038059)
Supplement: Table S3 — Primers used in S. sanguinis SK36. aAll primers were designed as part of this study. bUnderlined letters indicate restriction enzyme site. (DOC) [file pone.0038059.s004.doc]

**Table S3. Primers^a^ used in *S. sanguinis* SK36**

| **Primer** | **Sequence (5' to 3')^b^** |
| --- | --- |
| SK36A1F | ACGTCCGGTACCTTCTTGATTCCTGCCAATCC |
| SK36A1R | ACGTCCGAATTCCAGCTAATGCGGCTTCCTAC |
| SK36A2F | ACGTCCGGATCCAGCATGGTATAGCCGTCACC |
| SK36A2R | ACGTCCGAGCTCATGAGAACGGACAACCCTTG |
| SK36B1F | ACGTCCGGTACCAACATCAGCAGGGTCAATCC |
| SK36B1R | ACGTCCGAATTCCCCAACCGTCTCAATCAACT |
| SK36B2F | ACGTCCGGATCCCACTGCCATTCTCGTCTTGA |
| SK36B2R | ACGTCCGAGCTCACTCAGCTGCCTGATTTGGT |
| SK36C1F | ACGTCCGGTACCGGTATCAGCGAAATGCCAGT |
| SK36C1R | ACGTCCGAATTCGGCAAGGAGATTGATCCAAA |
| SK36C2F | ACGTCCGGATCCCATGCCTGCTGTCTCGATTA |
| SK36C2R | ACGTCCGAGCTCTCGAGAAGGCCAAGAAAGAA |
| SK36D1F | ACGTCCGGTACCAGGATTTGACAACGGTCAGG |
| SK36D1R | ACGTCCGAATTCATCCTCAGCTGCAGCAATTT |
| SK36D2F | ACGTCCGGATCCGAAAACAGCGCTTGAAAAGG |
| SK36D2R | ACGTCCGAGCTCCGCATCTGTCACCGTCTTTA |

*^a^* All primers were designed as part of this study.

*^b^* Underlined letters indicate restriction enzyme site.
