# Supplementary material for: Ecto-5′-Nucleotidase: A Candidate Virulence Factor in Streptococcus sanguinis Experimental Endocarditis
Source: PLoS One. 2012 Jun 7;7(6):e38059. doi: 10.1371/journal.pone.0038059 (PMC3369921; doi:10.1371/journal.pone.0038059)
Supplement: Table S4 — Primers used in S. sanguinis 133-79. aAll primers were designed as part of this study. bUnderlined letters indicate restriction enzyme site. (DOC) [file pone.0038059.s005.doc]

**Table S4. Primers^a^ used in *S. sanguinis* 133-79**

| **Primer** | **Sequence (5’ to 3’)^b^** |
| --- | --- |
| 133-79A1F | ACGTCCGGTACCAGCAAAGGCCACATCCATAG |
| 133-79A1R | ACGTCCGAATTCCGGTGGTTACCTGCAAATCT |
| 133-79A2F | ACGTCCGGATCCATGGTTTCCGACTGCCATAG |
| 133-79A2R | ACGTCCGAGCTCAAAAGGCGTTATCGGTGATG |
| 133-79ACom_F | ACGTCCGTCGACCTCTGATGAATTTCAGAGTC |
| 133-79ACom_R | ACGTCCGAGCTCCTAGTCTTCTTTATGACTTT |

*^a^* All primers were designed as part of this study.

*^b^* Underlined letters indicate restriction enzyme site.
